# Acoustic and phonemic processing are impaired in individuals with aphasia

**DOI:** 10.1038/s41598-023-37624-w

**Published:** 2023-07-11

**Authors:** Jill Kries, Pieter De Clercq, Robin Lemmens, Tom Francart, Maaike Vandermosten

**Affiliations:** 1 Experimental Oto-Rhino-Laryngology, Department of Neurosciences, Leuven Brain Institute, KU Leuven, Leuven, Belgium; 2 Experimental Neurology, Department of Neurosciences, KU Leuven, Leuven, Belgium; 3 Laboratory of Neurobiology, VIB-KU Leuven Center for Brain & Disease Research, Leuven, Belgium; 4 Department of Neurology, University Hospitals Leuven, Leuven, Belgium

**Keywords:** Auditory system, Stroke, Cognitive neuroscience, Language

## Abstract

Acoustic and phonemic processing are understudied in aphasia, a language disorder that can affect different levels and modalities of language processing. For successful speech comprehension, processing of the speech envelope is necessary, which relates to amplitude changes over time (e.g., the rise times). Moreover, to identify speech sounds (i.e., phonemes), efficient processing of spectro-temporal changes as reflected in formant transitions is essential. Given the underrepresentation of aphasia studies on these aspects, we tested rise time processing and phoneme identification in 29 individuals with post-stroke aphasia and 23 healthy age-matched controls. We found significantly lower performance in the aphasia group than in the control group on both tasks, even when controlling for individual differences in hearing levels and cognitive functioning. Further, by conducting an individual deviance analysis, we found a low-level acoustic or phonemic processing impairment in 76% of individuals with aphasia. Additionally, we investigated whether this impairment would propagate to higher-level language processing and found that rise time processing predicts phonological processing performance in individuals with aphasia. These findings show that it is important to develop diagnostic and treatment tools that target low-level language processing mechanisms.

## Introduction

Aphasia is an acquired language disorder that frequently occurs after a cerebrovascular accident (CVA), or stroke. Given that a stroke can impact diverse brain areas to a varying amount, the symptoms and severity of aphasia are heterogeneous, encompassing difficulties across all speech processing levels, e.g., acoustic, phonological, semantic or syntactic, and language modalities, e.g., speech comprehension, production, reading or writing^1,2^. Aphasia research mostly covers the assessment of higher-level language functions such as phonology, semantics or syntax^2,3^. Behavioral tests of lower-level comprehension functions, such as auditory spectro-temporal processing, are not part of the clinical test protocol^4–6^ and these aspects have been researched rather sparsely^7–10^. In particular, spectro-temporal processing of acoustic aspects, e.g., dynamic amplitude changes (such as rise times), has only been assessed in aphasia in two case studies^7,8^, even though it is crucial for speech understanding^11–14^. Spectro-temporal processing of phonemic aspects, e.g., dynamic frequency changes that help identifying phonemes, has been found to be impaired in individuals with aphasia (IWA)^15,16^. While there is some evidence that phonemic processing correlates with higher-level linguistic measures^15,17,18^, other studies have failed to see such a link^19,20^. Thus, the link between phonemic processing and higher-level language processing is not yet clear. Moreover, to date the link between dynamic amplitude processing and higher-level language measures has not yet been examined. Therefore, we investigated (1) whether a significant difference can be found between IWA and healthy controls based on rise time processing and phoneme identification tasks and (2) whether these tasks can predict higher-level phonological processing performance in IWA.

### Rise time processing

Auditory processing of dynamic changes in the amplitude of speech is crucial for segmenting speech into meaningful sublexical and lexical units and thus, for speech comprehension^13,14,21,22^. In fact, one of the most important auditory cues for speech comprehension is the envelope, i.e., amplitude changes over time^11,14^. Shannon et al.^11^ found that listeners can understand speech based on the temporal envelope alone. Further, Oganian and Chang^14^ investigated what landmark of the envelope would be encoded strongest in the superior temporal gyrus and demonstrated that it is the rate of amplitude change at acoustic onsets of the envelope, rather than the absolute amplitude. Processing of the rate of positive change (rise) in amplitude, here referred to as rise time, can be measured via a rise time discrimination (RTD) task. Efficient processing of rise time is important for identifying the onset of a phoneme or a syllable and hence, aids speech segmentation. Furthermore, the rise time gives information about the syllable stress, since stressed syllables have steeper rise times than unstressed ones^14,21,22^. Hence, rise time processing is crucial for comprehension.

Biedermann et al.^23^ observed that patients with a lesion in the right or left auditory cortex (due to middle cerebral artery damage), as well as patients with a lesion in the subcortical auditory structures, showed auditory processing impairments in signal discrimination tasks in noise targeting frequency, amplitude and duration aspects. The middle cerebral artery supplies the insular and auditory cortex (among other areas) with oxygenated blood^24^. An estimated 70% of stroke-induced aphasia cases result from a stroke that involves the middle cerebral artery to some extent^25,26^, hence an auditory processing impairment in a considerable number of IWA can be expected. Despite this fact and the importance of efficient dynamic acoustic processing of the amplitude for speech comprehension, the aphasia literature has largely neglected studying this feature of speech processing. Two case studies with each an individual with a left-hemispheric lesion found impaired detection of amplitude modulations^7,8^. However, processing of the rate of change of amplitude modulations has not yet been systematically investigated in aphasia in a larger sample size - to our knowledge. Nonetheless, some studies in IWA have focused on other, non-dynamic acoustic cues, such as gap or stimulus duration^9,27–29^. These studies found impaired processing of gap and stimulus duration changes in IWA compared to healthy controls in a variety of tasks, i.e., stimulus (tone or vowel) duration judgement tasks^27,28^, temporal order detection tasks based on gap duration changes^9^ and gap detection tasks^28,29^.

Assessment of RTD performance in children and adults with developmental dyslexia has shown that they performed significantly lower than healthy controls^30,31^. This task can thus be seen as a behavioral marker of dynamic acoustic processing deficits that seem to underlie phonological problems in individuals with reading difficulties (i.e., developmental dyslexia). In the present study, we investigate whether RTD performance can also present a behavioral marker of dynamic acoustic processing impairments in individuals with post-stroke aphasia.

### Phoneme identification

Although efficient rise time processing supports identification of speech sounds (i.e., phonemes), efficient processing of dynamic changes in frequency is also required. In order to identify phonemes regardless of speaker-related acoustic variability, e.g., variation in accent, speed or syllable stress, certain acoustic cues need to be inhibited to identify the correct phoneme category^32^. Phoneme identification is often overlooked in aphasia assessments. Especially spectro-temporal aspects of phoneme identification, such as processing of formant transitions (i.e., spectro-temporal changes in the speech signal defined by vocal tract movements), are rarely assessed in IWA. Phoneme identification can be assessed by presenting ambiguous speech sounds between two similar phonemes, e.g., /bA/ and /dA/^33^. This task offers a measure of how consistently speech sounds with some acoustic variation are classified into the same category and thus, how clearly defined the borders of the phonemic representations are^32,33^.

Functional magnetic resonance imaging (fMRI) research has shown that phoneme identification is localized in the left superior temporal gyrus and sulcus^34–36^. In IWA after stroke, it is not uncommon that the lesion coincides with this area supplied mostly by the middle cerebral artery^23,25^. Accordingly, phoneme processing has been investigated in aphasia in the past, specifically via phoneme identification or discrimination tasks^9,15–20,37–43^. On phoneme identification tasks specifically, the literature reports for the most part decreased performance in IWA compared to healthy controls as well as compared to stroke patients without aphasia^15,16,19,38^. Two of these studies have investigated phoneme contrasts that differed in frequency changes over time^15,16^, while the other studies investigated phoneme contrasts that purely relied on temporal differences, i.e. differences in voice onset time, and did not assess processing of dynamic spectro-temporal cues (formant transitions), which are important for phoneme identification. In an aphasia case study, Saffran et al.^15^ observed imapired processing of phoneme contrasts (stop consonants) that differed in manner of articulation and voicing. Gow et al.^16^ explored phoneme contrasts that differed in phonetic features (e.g., manner and place of articulation, voicing) in 22 IWA and observed that phoneme identification was impaired in IWA compared to healthy controls. Interestingly, the authors found larger impairments on phoneme contrasts that relied on the place of articulation than on voicing^16,18^. While voicing was experimentally manipulated by shifting the voice onset time, the phoneme contrasts relying on place of articulation require processing of dynamic frequency modulations (formant transitions). Given the involvement of this mechanism for speech comprehension, information about the consistency of identifying speech sounds into the same phonemic category may be important for diagnosis and therapy of speech processing problems in aphasia.

### Association between acoustic-phonemic processing and higher-level speech processing

Assessing acoustic and phonemic processing to inform therapy plans would be particularly helpful if a relation with higher-level speech processing performance would be present in aphasia. Neural models show that different speech processing mechanisms overlap in time and that the lower-level auditory analysis interacts bidirectionally with top-down contextual information to aid quick access to semantic representations^44,45^. Hence, the interaction between these systems might be adversely affected if one of the systems is defective. To explore the link between lower- and higher-level mechanisms, it has been investigated whether an impairment at lower-level speech processing would affect higher-level speech processing. In individuals with developmental dyslexia, evidence suggests that impairments in auditory spectro-temporal processing, measured via rise time discrimination and phoneme identification tasks, can propagate onto higher-level mechanisms, such as phonological processing and literacy measures^46–52^. In aphasia research, the link between phoneme identification and higher-level measures has been explored^15,16,18,19^, but the link between rise time discrimination and linguistic measures has not yet been investigated to our knowledge. With respect to phoneme identification tasks, Basso et al.^19^ did not find a link with performance on the token test, i.e., a test assessing comprehension of auditory instructions. However, auditory discrimination of stationary stimuli in IWA has been linked to phonological processing performance^9,10,17,28^. Furthermore, Robson et al.^10^ found a link between dynamic frequency modulation processing and comprehension and phonological tests in IWA.

To date, it is not yet clear whether rise time processing is associated with phonological processing in IWA and the link between phoneme identification and phonological processing could benefit from further research. Should such associations be present in aphasia, then targeting these acoustic and phonemic properties in interventions for aphasia could potentially result in a cascading effect on higher-level linguistic processing aspects.

### Current study

Our primary aim was to explore acoustic and phonemic processing in IWA and a control group by administering the RTD task and the phoneme identification task. The RTD task is a measure of (non-linguistic) auditory processing targeting dynamic changes in amplitude. The phoneme identification task indicates how well the phoneme boundaries are defined, based on subtle spectro-temporal changes in syllable-level stimuli. Both tasks are presented auditorily and thus require functional peripheral hearing, which is often impaired in older adults, such as the ones included in our study. We therefore statistically controlled for the influence of hearing levels. Furthermore, the two tasks also involve cognitive processes, such as attention and executive functioning. IWA frequently show concomitant cognitive impairments and thus, we also statistically controlled for the influence of cognitive functioning. We hypothesized IWA to show lower performance at group-level than the control group on the RTD and the phoneme identification task. To explore how many IWA would deviate from the control group, hence display impaired performance on the acoustic and phonemic tasks, we implemented an individual deviance analysis^53^. The second aim was to investigate whether the performance at the acoustic and phonemic tasks would predict performance at higher-level phonological tasks within the aphasia group. We expected scores on the acoustic-phonemic tasks to predict performance on the phonological processing tests.

## Methods

We compared acoustic and phonemic processing in IWA and age-matched healthy controls via two pyschoacoustic tasks, i.e., the RTD task and the phoneme identification task. To explore whether acoustic and phonemic processing skills in IWA are associated with higher-level phonological impairments, we also administered two language tests that measure phonological processing. Furthermore, a validated picture-naming task and a general aphasia test were administered to characterize the aphasia sample.

### Participants

We tested 29 IWA in the chronic phase ($\ge$ 6 months) after stroke (time since stroke in months: mean = 38.8, standard deviation (SD) = 70.7, median = 18, min = 6, max = 368) and 23 healthy age-matched controls. All participants were Dutch native speakers from Flanders, Belgium. IWA were recruited in two ways. Between October 2018 and March 2021 (with a COVID-19-related break between March and June 2020), patients at the stroke unit of the university hospital (UZ Leuven) were systematically screened for language deficits on a daily basis using the Language Screening Test (LAST)^54^. For this, informed consent was obtained from all screened patients, which was in accordance with the declaration of Helsinki. The study received ethical approval by the medical ethical committee of KU Leuven and UZ Leuven. Patients with a stroke that scored equal to or below the cut-off score were contacted earliest 6 months after the stroke to participate in the study. They also had to meet further inclusion criteria before they were contacted, i.e., having no formal diagnosis of a psychiatric or neurodegenerative disorder, and having a left-hemispheric or bilateral lesion. The second recruitment strategy for IWA encompassed contacting independent speech-language pathologists (SLP) and rehabilitation centra in Flanders to advertise the study via flyers and posters (see fig. S.1 for a flowchart of the recruitment strategies). Healthy age-matched controls (n = 23) were recruited via flyers positioned in recreational community centers for elderly. The accepted age for participation of healthy controls was gradually adapted based on the mean age and SD of IWA included in the study.

The resulting aphasia sample from the two recruitment strategies was checked for language impairments using two standardized diagnostic aphasia tests, i.e., the ScreeLing^55^ and the Dutch naming test (Nederlandse Benoem Test (NBT))^56^. The ScreeLing test does not include a picture-naming task, therefore we added the NBT to characterize aphasia. The NBT is a validated picture-naming test with 92 items and a maximum score of 276 points (cut-off threshold: 255)^56^. The ScreeLing is a validated test for diagnosis and therapy follow-up that consists of three subtests, i.e., phonology, semantics and syntax, each containing four tasks with 24 items^55^. Here, we administered the ScreeLing on a tablet using the Gorilla Experiment Builder (http://www.gorilla.sc)^57^. To check language impairments among IWA, we used the total score of the ScreeLing (maximum score: 72, cut-off threshold: 68). The test scores of IWA can be found in Table 1 and they are visualized in supplementary figure S.2. Note that no patient was excluded based on severity of language impairment measured on these tests. In fact, we included individuals that scored either (1) below the cut-off threshold on at least one of these two tests at the moment of data collection (n = 20) (Table 1), or (2) had a documented language impairment in the acute phase (8 of the 9 remaining individuals scored below cut-off threshold in the acute phase on the ScreeLing (n = 5), the Comprehensive Aphasia Test-NL (CAT-NL) (n = 2) or the Aachen Aphasia Test (n = 1) and one IWA, who was referred to the study via flyer, provided medical proof of a diagnosis of severe motor aphasia in the acute phase). Also note that 8 out of the 9 IWA, who did not score below the cut-off thresholds on neither the ScreeLing nor the NBT at the time of data collection, were still following speech-language therapy at the time of data collection (Table 1). Informed consent was obtained from all participants (or their legal guardian) prior to participation and this part of the study also got ethical approval by the the medical ethical committee of KU Leuven and UZ Leuven.

IWA were on average 71.52 years old (SD: 12.15) and controls were 71.52 years old (SD: 7.15). No age difference was found between groups (W = 365.5, *p* = 0.56) (supplementary fig. S.2). The sex ratio was not significantly different between both groups ($\chi ^2$= 3.4e-31, df = 1, *p* = 1; IWA: 27.6% female, 72.4% male; controls: 30.4% female, 69.6% male). The level of education did also not differ between groups ($\chi ^2$= 5.101, df = 4, *p* = 0.277; supplementary table S.1). The groups significantly differed on the NBT (W = 72, *p* < 0.001) and on the ScreeLing (W = 121, *p* < 0.001), as we expected given the inclusion criteria (supplementary fig. S.2). These variables and more demographic information (time since stroke, speech-language therapy) as well as lesion information (stroke type, blood vessel blocked or ruptured, lesioned hemisphere) about the aphasia sample can be found in Table 1. Note that out of the IWA of whom we had access to lesion information, 81.8% had a lesion in the middle cerebral artery. We did not have access to lesion information of 3 IWA and 4 IWA had bilateral lesions. We conducted separate analyses on these 7 IWA and compared them to the control group. The pattern of results was identical to the results of the main analysis that will be addressed in the following sections.

**Table 1:** Demographics and lesion information of the aphasia group.

ID (n = 29)	Age	Sex	Time since stroke (months)	Stroke type	Blood vessel	Lesioned hemi- sphere	SLT	NBT score (max = 276, cut-off = 255)	ScreeLing score (max = 72, cut-off = 68)
sub-006	86	m	22.8	Ischemia	VA	Bilateral	Yes	263	70
sub-008	71	f	21.1	Ischemia	MCA	Left	No	273	69
sub-009	67	m	22.6	Ischemia	MCA	Left	No	262	65.5
sub-014	75	m	18.2	Ischemia	MCA	Left	Yes	185	58
sub-016	68	m	18.0	Ischemia	MCA	Bilateral	Yes	265	69
sub-017	88	m	8.2	Ischemia	MCA/ PCA	Bilateral	No	245	56
sub-018	61	f	29.4	Ischemia	MCA	Left	Yes	263	72
sub-019	72	m	8.6	Ischemia	PCA	Left	–	211	51
sub-020	42	m	18.7	Ischemia	MCA	Left	Yes	273	71
sub-021	81	m	8.6	Hemorrhage	–	Left	No	252	70
sub-022	78	f	8.2	Hemorrhage	–	Left	Yes	207	56
sub-023	90	m	22.8	Ischemia	LLA	Bilateral	No	265	61
sub-024	69	m	6	Ischemia	MCA	Left	Yes	260	68.5
sub-025	69	m	11.5	Ischemia	MCA	Left	Yes	197	58.5
sub-026	71	m	31	Ischemia	MCA	Left	Yes	250	69
sub-027	76	m	126.2	Ischemia	MCA	Left	Yes	254	63
sub-028	80	m	25.2	Ischemia	–	Left	No	195	54
sub-029	75	m	22.6	Ischemia	MCA	Left	Yes	193	55
sub-030	49	m	13.1	Hemorrhage	–	Left	Yes	217	57
sub-031	79	m	12.9	Ischemia	MCA	Left	Yes	263	63
sub-032	76	m	94.8	Ischemia	MCA	Left	Yes	3	28
sub-034	79	m	13.4	–	–	–	Yes	244	69
sub-035	64	f	31.5	Ischemia	MCA/ ACA	Left	Yes	276	71
sub-038	60	f	368.6	–	–	–	No	251	65
sub-049	85	f	8.3	Ischemia	PICA	Left	Yes	242	65.5
sub-050	81	f	8.7	Ischemia	MCA	Left	Yes	134	53
sub-052	72	m	120.7	–	–	–	Yes	175	48
sub-053	41	f	6.1	Ischemia	MCA	Left	Yes	271	70
sub-054	69	m	17.2	Ischemia	MCA	Left	Yes	268	69
Total	–	21 male 8 female	–	23 ischemia 3 hemorrhage	18 MCA 2 PCA 1 ACA 1 VA 1 LLA 1 PICA	22 left 4 bilateral	21 yes 7 no	12 above 17 below cut-off	12 above 17 below cut-off

SLT: speech-language therapy; NBT: Dutch naming test (Nederlandse Benoem Test); VA: vertebral artery; MCA: middle cerebral artery; PCA: posterior cerebral artery; ACA: anterior cerebral artery; LLA: lateral lenticulostriate arteries; PICA: posterior inferior cerebellar artery; –: data not available.

### Behavioral measures used for statistical analyses

#### Rise time discrimination task

**Figure 1:**
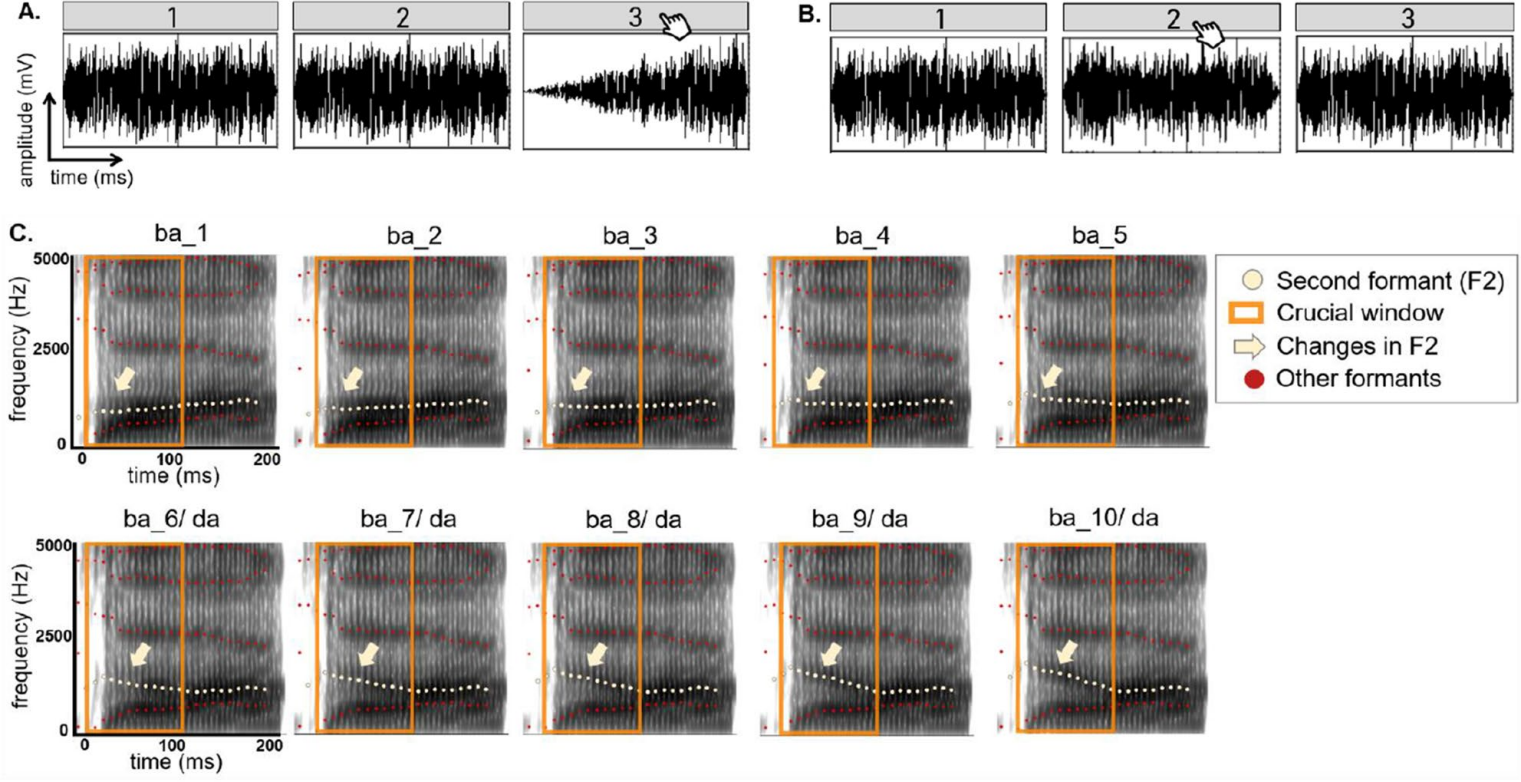
Stimuli used for the auditory-phonemic tasks. (**A**) Visualization of the RTD task with an example of the largest rise time contrast between the reference stimuli (15 ms rise time) and the deviant stimulus (699 ms rise time). The deviant stimulus position was randomized across trials. (**B**) Visualization of the RTD task with an example of the smallest rise time contrast between the reference stimuli (15 ms rise time) and the deviant stimulus (28 ms rise time) identified by the highest performing participant in this study. In (**A**) and (**B**) the deviant stimulus is indicated by the hand cursor. (**C**) Visualization of the 10 stimulus steps used for the categorical perception task. The yellow dots depict F2, the yellow arrow points to the change in the slope within the first 100 ms of each stimulus (orange square). After 100 ms, the stimuli remain unchanged. The other formants, depicted by the red dots, also remain unaltered across stimulus steps.

**Figure 2:**
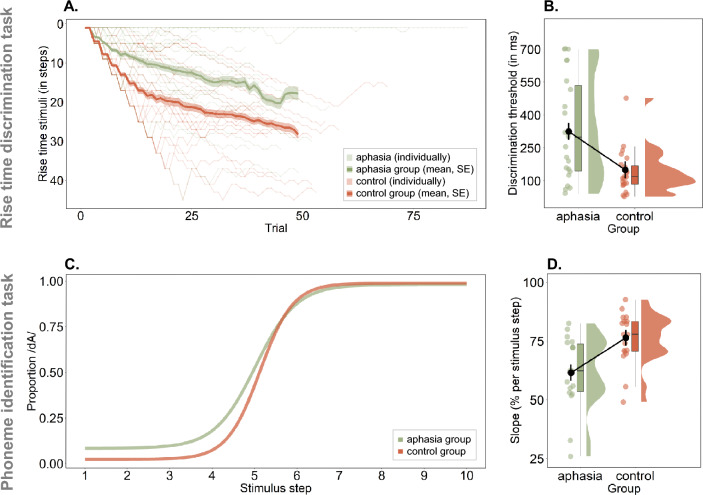
IWA show decreased performance at two auditory-phonemic processing tasks. (**A**) The RTD thresholds were determined via a one-up/two-down adaptive staircase procedure. This figure shows the individual performance trajectories as well as the group average and standard error across trials for visualization purpose. The deviant stimulus rise times of the last 4 reversals were averaged to determine the final threshold, which was used for statistical analysis. (**B**) IWA at group level processed the rate of change in amplitude less precisely (i.e., larger differences in rise time are needed to discriminate stimuli) than healthy, age-matched controls, even when controlling for hearing and cognition. (**C**) Visualization of the average of subject-specific psychometric functions by group, representing performance on the phoneme identification task. The steeper the slope, the more consistently a phoneme is identified, hence suggesting better phoneme representation. (**D**) IWA showed significantly lower slopes than age-matched controls at group level on the phoneme identification task, even when controlling for hearing and cognition.

The RTD task measures how well participants discriminate the rate of change in amplitude at the onset of a sound. Precisely, the task was presented as a three-alternative forced choice task, where the deviant stimulus had to be discriminated from two identical reference stimuli (Fig. 1A,B). The stimuli were created in MATLAB^58^ using one-octave noise bands centered at 1 kHz^31^. The software APEX was used to present the task^59^. Stimuli were calibrated and presented in the left ear at 70 dB SPL. The reference stimulus had a rise time of 15 milliseconds (ms). The deviant stimuli were computed to have rise times that decreased logarithmically in 50 steps from 699 ms to 16 ms. The duration of each stimulus was 800 ms. The number of trials differed between participants, as the task followed a one-up/two-down adaptive staircase procedure. This means that after two correct responses in a row, the difference in rise time between stimuli became smaller, thus more difficult, during the next trial. After one erroneous response, the difference in rise time between stimuli became larger, thus easier to discriminate. This way, a threshold corresponding to 70.7% correct was targeted^60^. The task ended once 8 reversals (i.e., changes in direction) were reached. In case no reversals were present, the task ended after a maximum of 87 trials. The individual performance trajectories as well as the group average and standard error (SE) of the trajectories are visualized in Fig. 2A. The rise times of the deviant stimulus of the last 4 reversals were averaged to determine the final threshold. This threshold was used for statistical analyses.

To make sure that the task was well understood by all participants, they performed between 4 and 8 practice trials before starting the task. In the aphasia group, 23/29 IWA completed the RTD task, while 6 IWA experienced the task as too difficult after the initial trials. All 23 healthy controls completed the task. Statistical analyses involving this test were thus performed on 23 IWA and 23 healthy controls.

#### Phoneme identification task

The phoneme identification task assesses how consistently speech sounds (here /bA/-/dA/) are identified. We used the same task and stimuli as employed in Vandermosten et al.^33^. The task was presented as a two-alternative forced choice identification task. Participants were instructed to decide whether the stimulus they heard sounded more like a /bA/ or more like a /dA/. The stimuli were created based on a naturally spoken /bA/. The first 100 ms of the second formant (F2) of this syllable was linearly interpolated in 10 steps to create the stimuli, using Praat (Praat^61^; see Vandermosten et al.^33^ for more details). The difference between /bA/ and /dA/ solely relies on the F2 slope, this way a gradual continuum was created between these speech sounds (Fig. 1C). Thus, distinguishing between the two speech sounds relies mostly on dynamic cues, namely the discrimination of the spectral changes of F2 over time (i.e., whether the F2 slope is rising or falling). During the task, each of the 10 stimulus steps was presented 8 times in a randomized order, i.e., 80 trials. At the start of the task, the two speech sounds at the extremities of the stimulus spectrum were presented as reference practice trials. The stimuli were calibrated and presented monaurally at 70 dB SPL. The software APEX was used to present the task^59^.

The amount of /dA/ responses for each stimulus step was taken and divided by 8 (i.e., number of presentations per stimulus step), to arrive at the proportion of /dA/ responses. This allowed us to fit a psychometric curve on the data points using the toolbox Psignifit in MATLAB (https://github.com/wichmann-lab/psignifit)^58^. This toolbox allows to fit subject-specific guess and lapse rates, thereby we avoided making assumptions about performance at the extremities of the stimulus continuum, hence the slope was not affected by such assumptions. As borders for the guess rate, we defined a range between 0 and 0.89 and for the lapse rate a range between 0 and 0.1 on the scale of proportion of /dA/ responses. We used uniformly distributed priors in order to avoid biasing the definition of the lapse and guess rate. Figure 2C shows the psychometric curves averaged by group. Subsequently, the slope at the subject-individual 50$\%$ point was computed using the function getSlope from the same toolbox and was used for statistical analyses. It is an indicator of how consistently participants were able to categorize the stimulus steps, which is indicated by the steepness of the slope.

In the aphasia group, 26/29 IWA completed the phoneme identification task, while 3 IWA experienced the task as too difficult after some initial trials. All 23 healthy controls completed the task. As a quality check, the confidence intervals of the lapse and guess rates were analyzed. Participants whose confidence interval of either of the asymptotes included 0.5 on the y-axis (i.e., the proportion of /dA/ responses; Fig. 2C) were excluded from the analysis, i.e., 8 IWA and 4 healthy controls. Thus, all statistical analyses involving this test were performed on 18 IWA and 19 healthy controls.

#### Phonological higher-level tasks

In order to answer the second research question, i.e., the potential link between acoustic-phonemic tasks and phonological processing, we used two tests, i.e., phonological word fluency and the phonology subtest of the ScreeLing. We used these measures to check whether scores on the RTD and phoneme identification task would predict the performance of IWA on these phonological higher-level tasks (within aphasia group analysis).

##### Phonological word fluency 

We administered the phonological word fluency subscale of the CAT-NL^62^. Participants were required to enunciate as many words as possible that start with the letter ‘s’ within one minute. The score consisted of the number of correct words expressed. Phonological word fluency tasks require recruitment of linguistic functions, such as phonological processing and knowledge. However, note that phonological fluency tasks also involve cognitive functions, such as attention, executive functions and memory^63–66^. We therefore controlled the linear models for the influence of cognitive functions, namely attention, executive functions and memory, in addition to controlling for hearing function.

##### Phonology subtest of the ScreeLing

The phonology scale consists of four tasks, i.e., spoken word repetition, reading out loud, minimal pair discrimination and initial phoneme identification. The two first tasks of the phonology subtest require the participant to produce speech as an answer, whereas the latter two tasks are receptive, i.e., the participant has to point at the answer. Specifically, the minimal pair discrimination task was auditorily presented, i.e., 2 words followed by “were the two words you heard identical?”, and participants could point to yes or no or say it out loud. The initial phoneme identification task was presented simultaneously visually and auditorily, i.e., “what is the first letter of ’word’?”, in reply to which participants got 4 multiple choice options and they could point at the answer or say it. Each of the tasks consists of 6 items, hence the total score on the phonology subtest is 24.

#### Nuisance variables

We used measures of hearing and cognitive functioning as nuisance variables to take into account their potential influence on the dependent variable in the statistical models (“Section Statistical analyses”).

##### Hearing

Hearing thresholds were assessed via pure tone audiometry (PTA) at frequencies ranging from .25 to 4 kHz. The Fletcher index (average of thresholds at 1 , 2 and 4 kHz) was calculated per ear and subsequently averaged across both ears. The thresholds did not differ between IWA and healthy controls (t = 0.582, df = 49.499, *p* = 0.563).

##### Cognitive functions

We administered the Oxford Cognitive Screen-NL (OCS) as cognitive test^67^. This validated test was designed to be language-independent, such that cognitive functioning can be disentangled from language functioning. Here, we used the subscales attention (i.e., crossing out target shapes among distractor shapes), executive functions (i.e., connecting circles and triangles in alternation in descending order of size) and memory (i.e., free recall and recognition of words and shapes) to calculate a composite score of cognition. The aphasia group had significantly lower cognitive scores than the healthy control group (t = − 4.905, df = 33.759, *p* = < 0.001).

### Statistical analyses

Statistical analyses were performed in R^68^. We used parametric tests and then checked whether the normality assumptions were met (supplementary table S.2). If this was not the case, we conducted and reported non-parametric tests.

#### Research question 1

 A two-tailed, unpaired Student’s t-test was performed to analyze group differences on the RTD and a two-tailed, unpaired Wilcoxon test to analyze differences on the phoneme identification tasks between the aphasia group and the healthy control group. The scores on the RTD task were log-transformed for statistical analyses because the outcome scores were logarithmically distributed, which was expected given the nature of the stimuli. Both the RTD and the phoneme identification task were auditorily presented and thus, require functional peripheral hearing. Older adults, independent of having aphasia or not, are prone to age-related hearing loss (i.e., presbyacusis)^69^. To account for individual differences in age-related hearing loss across all participants, we statistically controlled for its influence in a second step of the group comparison analysis. Furthermore, the two tasks also involve cognitive processes, such as attention and executive functioning. IWA frequently show concomitant cognitive impairments and thus, we also statistically controlled for the influence of cognitive functioning differences. Thus, to see whether the group effect would uphold when controlling for these variables, we added hearing levels (i.e., the Fletcher index) and cognition (i.e., composite score of OCS subtests of attention, executive functions and memory) to the models. We did not introduce interaction effects between group and hearing or cognition in the model, as we were only interested in the main effect of group. For the linear model used for the RTD task, we used the following syntax: *task scores* $\sim$ *group + hearing + cognition*. For the phoneme identification task we used a generalized additive model. We first checked which independent variables used more than one base function (i.e., is non-linear), which was hearing, and then applied the following syntax: *task scores* $\sim$ *group + s(hearing) + cognition*.

An individual deviance analysis, as described in previous literature^49,53,70^, was performed on the RTD and phoneme identification task. In essence, for this analysis a reference distribution was created based on a trimmed control group, and IWA were considered to deviate from this norm when their score exceeded 1.65 SD. More specifically, in a first step the lowest performing 5$\%$ of the control group were removed from the control group, which will be referred to as trimmed control sample. The mean and SD of the trimmed control sample were then used to standardize the raw task scores of all participants (IWA and all healthy controls). The deviance threshold was then defined at 1.65 SD for the RTD task and for the phoneme identification task at -1.65 SD of the z-scored distribution. Scores below the deviance threshold were viewed as deviant from the control sample (see supplementary material for more details on the implementation).

#### Research question 2

To investigate whether performance on the acoustic and phonemic tasks would predict performance at phonological processing tasks in IWA, we employed a linear model with the following syntax: *phonological task scores* $\sim$ *acoustic-phonemic task scores + hearing + cognition*. We again controlled for the influence of hearing and cognition. An ANOVA was performed to test predictors of these linear models. The *p* values were corrected for multiple comparisons (n = 2, i.e., 2 phonology-level tests) using the false discovery rate (FDR) method^71^.

## Results

### Research question 1: Comparison of the aphasia group with the healthy control group

We compared the RTD threshold of the aphasia group to the healthy control group and found a significant group difference (p = 0.001) (Fig. 2B). All results are shown in Table 2. IWA displayed on average a larger RTD threshold than healthy controls, meaning that they needed larger differences in rise time between the reference stimulus (rise time of 15 ms) and the deviant stimuli for discrimination. The group difference remained significant even after controlling for hearing and cognition (p<0.001) (Table 2).

We also compared the phoneme identification slopes of the aphasia group to the healthy control group. We found a significant group difference (p = 0.006) (Fig. 2D, Table 2). IWA displayed on average less steep slopes of the psychometric function fitted to their data than healthy controls (Fig. 2C), meaning that they did not classify the speech sounds as consistently in the same category as healthy controls. The group difference remained significant when controlling for inter-individual variability in hearing and cognition (*p* = 0.004). Neither hearing nor cognition significantly contributed to the model (Table 2).

#### Individual deviance analysis

We analyzed whether each IWA was deviant from the control group on the RTD and phoneme identification task. The original control group (n = 23) was trimmed by removing two controls for the RTD task and one for the phoneme identification task, resulting in trimmed control samples of 21 and 22 participants respectively (see supplementary material for more details). After standardization of the scores based on the trimmed control group, we found that 12 out of 23 (52.2$\%$) IWA were deviant from healthy controls on the RTD task. 10 out of 18 (55.5$\%$) IWA were deviant from healthy controls on the phoneme identification task (supplementary table S.3). The groups’ distributions after standardisation of the scores relative to the deviance threshold are visualized in supplementary figure S.4. Taking the two tasks together, in total 19 out of 25 IWA (76$\%$) were deviant on at least one of the tasks, meaning that three quarters of the aphasia sample had an impairment on at least one of the two auditory lower-level processing tasks.

### Research question 2: Relation between acoustic-phonemic processing performance and phonological processing

**Figure 3:**
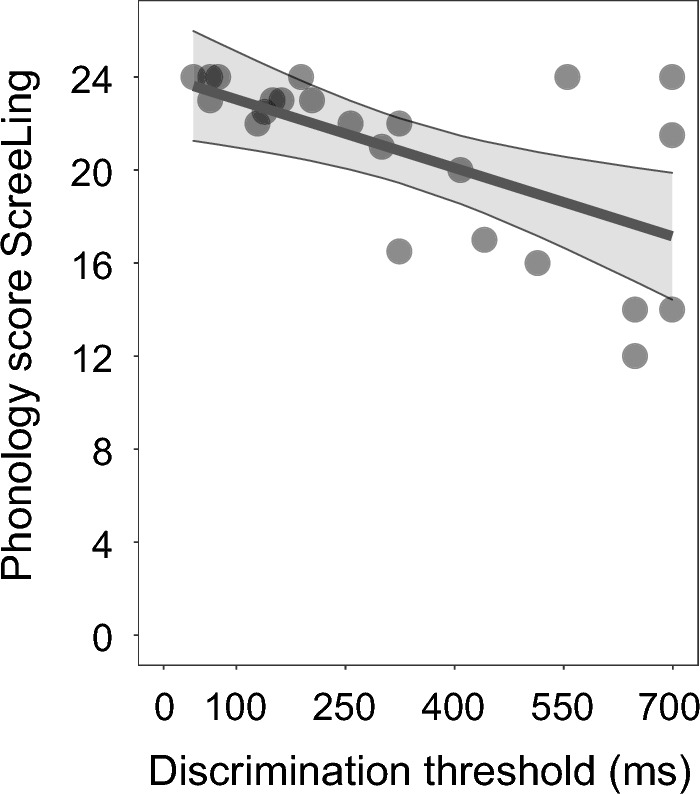
Performance on the RTD task predicts performance on the phonology subtest of the ScreeLing within IWA. IWA that detected differences in rise times only when the difference was relatively larger between standard and deviant stimuli (=lower performance) also scored lower on the phonology subtest of the ScreeLing, which consists of a composite score of four tasks, namely spoken word repetition, reading out loud, minimal pair discrimination and initial phoneme identification.

We investigated whether RTD scores would predict the outcomes on the phonology subtest of the ScreeLing and the phonological word fluency test within the aphasia group. We found that the RTD thresholds significantly predicted scores on the phonology subtest of the ScreeLing (*p* = 0.006) (Table 2 and Fig. 3). The larger the RTD thresholds were in IWA (i.e., the lower the performance), the lower the score was on the phonology subtest. This effect was present even though we controlled for inter-individual variability in hearing and cognitive functioning. Performance on the RTD task did not predict outcomes on the phonological word fluency task (*p* = 0.362). None of the factors we controlled for in the statistical model had a significant contribution to either of the models. Thus, the scores on the phonology ScreeLing subtest were predicted by the RTD scores above and beyond individual variations in hearing or cognitive functioning.

We also analyzed whether performance on the phoneme identification task would predict performance on the phonological processing tests within the aphasia group. We found that the phoneme identification slopes did neither predict scores on the phonology subtest of the ScreeLing (*p* = 0.265) nor on the phonological word fluency test (*p* = 0.989) within the aphasia group (Table 2).

**Table 2:** Results.

	Effect	adj.R^2^	F	t	W	DF	Beta estimates	Std. error	*p* value
*Rise time discrimination task*									
Group comparison without controlling	**Group**			3.43		41.82			**0.001**
Group comparison with controlling	**Model performance**	0.29	7.20			(3, 42)			< **0.001**
	**Group**		13.45			1	− 0.53	0.25	< **0.001**
	**Hearing**		4.82			1	0.009	0.007	**0.033**
	Cognition		3.31			1	− 0.02	0.01	0.075
Phonology ScreeLing (within-aphasia group)	Model performance	0.27	3.81			(3, 19)			0.052^b^
	**RTD task**		11.37			1	− 2.70	0.87	**0.006**^b^
	Hearing		0.08			1	0.01	0.04	0.778^b^
	Cognition		0.005			1	0.004	0.06	0.942^b^
Phonological word fluency (within-aphasia group)	Model performance	− 0.02	0.82			(3, 19)			0.498^b^
	RTD task		0.86			1	− 2.18	1.58	0.362^b^
	Hearing		0.95			1	0.04	0.08	0.682^b^
	Cognition		0.64			1	− 0.08	0.11	0.864^b^
*Phoneme identification task*									
Group comparison without controlling^a^	**Group**				81.5				**0.006**
Group comparison with controlling^a^	Model performance	0.33							
	**Group**		9.39			1	14.87	4.85	**0.004**
	Hearing		2.51			(2.67, 3.34)*			0.071
	Cognition		0.88			1	− 0.21	0.22	0.352
Phonology ScreeLing (within-aphasia group)	Model performance	0.35	4.07			(3, 14)			0.056^b^
	Ph. id. task		2.54			1	− 0.08	0.04	0.265^b^
	**Hearing**		7.68			1	− 0.06	0.05	**0.030**^b^
	Cognition		1.99			1	0.06	0.04	0.36^b^
Phonological word fluency (within-aphasia group)	Model performance	− 0.18	0.12			(3, 14)			0.944^b^
	Ph. id. task		0.0002			1	0.04	0.13	0.989^b^
	Hearing		0.31			1	0.10	0.18	0.583^b^
	Cognition		0.05			1	0.03	0.14	0.811^b^

adj.R^2^: adjusted R^2^; F: F-test value; t: t-test value; W: Wilcoxon test value; DF: degrees of freedom; Std. error: standard error; ^a^: non-parametric tests applied; ^b^: FDR-correction for multiple comparisons was applied (n = 2); *: effective DF and reference DF; significant effects are marked in bold.

## Discussion

We investigated acoustic and phonemic processing in individuals with post-stroke aphasia and age-matched healthy controls. Specifically, we administered two auditory tasks that rely on dynamic amplitude and spectral changes, which have not been investigated before in aphasia. The RTD task requires participants to detect small changes in amplitude. The phoneme identification task measures how consistently spectro-temporally varying, ambiguous speech sounds are classified in the same phonemic category and reflects how robust phoneme representations are (Fig. 1). Assessing these tasks in IWA thus allows us to gain knowledge about lower-level auditory processing mechanisms that are currently neither assessed in clinical practice, nor treated in therapy. Here we did find group differences on both tasks, demonstrating lower performance in the aphasia group in rise time processing and phoneme identification than in the control group, even after controlling for the influence of hearing levels and cognitive functioning (Fig. 2, table 2). We also observed that more than half of IWA were deviant from the control group on the RTD and on the phoneme identification task (supplementary fig. S.4). Taken both tasks together, three quarters of IWA were impaired at least at one of the tasks, and thus had an impairment at auditory spectro-temporal processing (supplementary table S.3).

Given that dyslexia research has found evidence for cascading effects from lower-level to higher-level processing impairments and aphasia research a link between auditory and phonological processing, we investigated whether performance of IWA on the acoustic and phonemic processing tasks can predict performance at two phonological processing tests, namely phonological word fluency and the phonology subtest of the ScreeLing (consisting of four tasks, i.e., spoken word repetition, reading out loud, minimal pair discrimination and initial phoneme identification). Indeed, we found that performance on the RTD task predicted performance on the phonology subtest of the ScreeLing, revealing that IWA who displayed lower performance on the RTD task also had lower scores on the phonology subtest of the ScreeLing (Fig. 3, table 2). However, performance on the phoneme identification task did not significantly predict scores at any of the phonological tests. We will discuss the implications of these findings in detail here below.

In the current study, dynamic acoustic processing, measured via the RTD task, was lower in IWA than in healthy controls. Rise time processing has never been tested before in IWA, but our results are in line with previous studies investigating acoustic processing via non-dynamic psychoacoustic experiments^9,27–29^. However, Oganian and Chang^14^ demonstrated that processing of amplitude envelope modulations at the onset of speech sounds, i.e., acoustic onset edges and their slope steepness, rather than the absolute amplitude (i.e., static), is a crucial cue for speech comprehension. Therefore, assessing the dynamic processing of the rise time in IWA provides more specific information about potential impairments in speech envelope processing than static amplitude discrimination tasks. Deficient processing of the rise time during real-life speech can have adverse effects on understanding speech, because of its contribution to parsing the continuous speech stream into sublexical and lexical segments^22^. Moreover, given that syllable stress influences the rise time^14,21,22^, deficient rise time processing may lead to impaired processing of the syllable stress and thus, it becomes harder to follow the speech prosody and to comprehend speech in an efficient way.

Here, we found that more than half of IWA had impaired rise time processing, hence demonstrating that the assessment of lower-level auditory processing in IWA is important. As a matter of fact, 6 of our participants with aphasia were not able to perform this task because it was too difficult, potentially exhibiting an even larger proportion of IWA to be impaired on this task. We did not inquire about the specific reasons why these participants refused to proceed with the task. While there is a chance that IWA may not have understood the task instructions, we believe that this is unlikely given that we introduced the task via practice trials. We believe that the stimuli were potentially too difficult for some participants, instead future studies could enlarge the rise times of the stimuli (e.g., to 1000 ms instead of 699 ms). This would be important to explore in the future, should the task be considered for diagnosis of lower-level auditory processing impairments in IWA. Overall, we suggest that being aware of an auditory processing impairment in a patient with aphasia could be useful for setting up an intervention plan targeting rise time processing and for following up on the recovery progress.

Execution of the RTD task also requires functioning sensory hearing and cognitive processes. Therefore, we statistically controlled for the variance explained by these factors in our analyses. The group effect remained significant. Nonetheless, we acknowledge that the administration of the RTD task would be difficult or impossible in patients with more severe conditions of cognitive impairment, motor impairments or hemineglect, which often occur in the acute phase after stroke, or in patients with severe hearing loss, thereby presenting a limitation for using the RTD task for diagnosing lower-level auditory processing impairments in aphasia at the person-specific level. This is also true for the phoneme identification task.

Also true for both the RTD and the phoneme identification task is that the age of the tested person may affect the performance. For instance, age-related differences in hearing and cognition may affect task performance. However, here the control group was age-matched to the aphasia group. Nonetheless, should the tasks be used to diagnose lower-level auditory processing impairments in the future, then norm scores should be developed in an age-specific manner. Future studies could investigate interaction effects between age and group. Processing speed may also have an influence on how well the dynamic acoustic and phonemic aspects are processed. Since IWA have lower domain-general processing speed than age-matched healthy controls^72,73^, it may be interesting in the future to also control for processing speed.

For efficient speech comprehension, certain cues of within- and between-speaker variability need to be inhibited in order to correctly identify phonemes^32^. The speech sounds used in the current phoneme identification task were artificially created to vary at different levels of ambiguity between /bA/ and /dA/, with the only difference between the sounds relying on spectro-temporal changes of the second formant within the first 100 ms after onset. In order to define the inter-category boundary between these phonemes and to consistently classify the same ambiguous stimuli into the same phoneme category, fine-tuned auditory spectro-temporal processing skills are essential. Thus, multiple processes are necessary for this task, i.e., sensitive auditory spectro-temporal processing, neglecting the variance within-speech sound category and linking the speech sound to phoneme representations in the brain (via interaction with long-term memory)^74–76^.

Our results revealed that IWA identify phonemes less consistently than healthy, age-matched controls. Thus, it seems that IWA at group-level have less robust phoneme representations. Same as for the RTD task, the group difference remained significant after controlling for the variance explained by hearing and cognitive functioning. The group difference result is in line with studies reporting decreased phoneme identification performance in specific subtypes and cases of aphasia^15,16,19,38^. Robson et al.^10^ did explore dynamic frequency modulations of non-speech sounds and also found decreased performance in individuals with severe Wernicke’s aphasia. The current study expands the previous findings to a more broadly recruited group of IWA, whose severity and type of aphasia is more heterogeneous.

Given the current data set, we cannot say whether an impairment in consistently identifying phonemes may be due to inefficient auditory spectro-temporal processing, or rather due to difficulties with neglecting within-phoneme category variance or with linking the sound to the correct phoneme representation^74–76^. Administering a phoneme discrimination task in addition to the phoneme identification task may be useful to disentangle the underlying processes in the future. The phoneme discrimination task requires participants to indicate whether two speech sounds are the same (^16,37,40^; also see supplementary experiment of Schevenels et al.^77^). Hence, categorizing phonemes is not necessary and a potential impairment on this task would be due to poor spectro-temporal processing, eliminating the possible influence of other processes. In future studies, we suggest to administer a phoneme discrimination task as a complement to the phoneme identification task to isolate the involved processes.

Not only did we find lower performance on the phoneme identification task in IWA, but we also detected that more than half of IWA have an impaired performance on this task, as evidenced by the individual deviance analysis. If we take into account the participants that had to be excluded from the analysis of the phoneme identification task (n = 8 IWA) because of too poor performance to fit a meaningful psychometric function, then 18 out of 26 IWA (69.23$\%$) were deviant from healthy controls on the phoneme identification task. The large proportion of IWA deviant on this task shows that it is important to assess phonemic processing in aphasia and train phonemic representations during the recovery process. This result is in line with findings of Robson et al.^10^, who also reported relatively high proportions of deviance in IWA on three dynamic frequency modulation tasks.

Taking a look at the overlap of deviance between the RTD task and the phoneme identification task in IWA, we saw that only a limited amount of them showed concordant deviance. In fact, 62.5$\%$ of IWA did not show an overlap of deviance between tasks, meaning that they were deviant on the RTD task but not on the phoneme identification task or vice versa. Even though both tasks require analysis of low-level auditory aspects, this shows that the two tasks do, at least partially, not measure one same construct of auditory spectro-temporal processing. While the RTD task assesses dynamic changes in amplitude, the phoneme identification task measures dynamic changes in frequency. Thus, it is possible that some IWA have more difficulties with processing amplitude changes, whereas others struggle more with dynamic changes in frequency. Still others might face difficulties in processing both aspects. However, given the small sample size in this study, we cannot draw strong conclusions. Nonetheless, it would be interesting to further investigate this in the future.

We also explored whether auditory processing would predict phonological processing in IWA. In the past, theoretical models of speech processing have viewed the different steps to be sequential and unidirectional, i.e., the auditory phonological analysis is followed by integration into the phonological lexicon, which is in turn followed by activation of the semantic system^78,79^. More recent models, however, show that different speech processing levels interact bidirectionally with each other^44,45^. In both types of models, an auditory processing impairment may propagate onto higher-level processes. Here, we tested this hypothesis and found that performance on the RTD task predicts higher-level phonological processing in IWA, as measured by the phonology subtest of the ScreeLing. This test analyzes phonological processing at a metalinguistic level, e.g., phonological awareness. The link between processing of dynamic amplitude modulations and phonological processing has not yet been investigated to our knowledge. However, using a different type of spectro-temporal processing task, Robson et al.^10^ reported an association between auditory processing of frequency modulations and the phonological discrimination task, which is thus in line with the current results. In individuals with developmental dyslexia, rise time processing has been found to predict phonological processing performance and literacy measures^46,47,49–51^. However, the underlying impairments in dyslexia and aphasia may differ, so future studies may want to reproduce our current finding in aphasia.

Integrating our findings, we established that more than half of IWA showed impaired performance on the RTD task and this performance relates to phonological processing. This could have interesting implications for diagnosing lower-level auditory processing impairments in IWA and for therapy of aphasia. Could therapy targeting improvement of rise time processing possibly show transfer effects on phonological processing in IWA? Taking a look at intervention research, a study in children at cognitive risk for dyslexia has shown that an intervention with enhanced envelope listening improved RTD performance^80^. In IWA, Szymaszek et al.^81^ showed that a training in temporal processing improved not only temporal processing performance, but also transferred to language comprehension tasks. Hence, having tools available to assess auditory processing in IWA paves the way for developing according treatment methods in the future, which may even show transfer effects to higher-level language processing.

In contrast to the RTD task, we found that the phoneme identification task did not predict performance at either of the phonological tests in IWA. The literature also reported ambiguous results on this matter, i.e., some studies found a link but others did not [see^15,17–20^]. Robson et al.^10^ found a link between frequency modulation processing and phonological processing in IWA, although in a small sample size and with a different auditory task. In dyslexia research, the phoneme identification task has been linked to higher-level phonological processing^48^, suggesting that deficits at the phonemic processing level do propagate onto higher-level speech processing mechanisms in dyslexia. The lack of such a result here might be due to different underlying impairment mechanisms in dyslexia and aphasia, due to the absence of a link between these processing steps in aphasia or it may be due to the small sample size here. Studies of aphasia with a larger sample size may shine light on this in the future.

Unlike the performance of IWA on the ScreeLing phonology subtest, their performance on the phonological word fluency test was not predicted by the rise time processing performance. We have two possible explanations for this result. First, we suggest that the amount and intensity of cognitive involvement is larger during the word fluency test than for the ScreeLing phonology subtest. The phonological word fluency task is time-constrained, attention-heavy and participants need to make use of executive functions, such as strategy formation, verbal memory (word retrieval), word knowledge and giving goal-directed responses according to the task rule^63–65^. Phonological word fluency is not solely used as a task to measure language performance, but also to measure executive dysfunction^64,65^. The ScreeLing phonology subtest on the other hand contains tasks requiring auditory attention, verbal short-term memory and decision-making processes. Thus, comparing the cognitive processes involved in these two tests, the word fluency test involves more and more costly mechanisms than the phonology subtest of the ScreeLing. Second, the cognitive processes involved in the RTD task, as well as the phoneme identification task, are more similar to the ones involved in the ScreeLing phonology subtest than to those involved in the word fluency test. The acoustic and phonemic tasks involve attention, short-term memory and decision-making. These cognitive processes are similar to the ones involved in the ScreeLing phonology subtest, but differ from those involved in the word fluency task.

A limitation of the current study is the rather small sample size. Even though we initially tested 29 IWA and 23 healthy controls, these sample sizes were reduced to 18 IWA and 19 controls on the phoneme identification task and to 23 IWA and 23 controls on the RTD task. Participants had to be excluded partly due to premature cessation of the task by the participants and partly due to the analysis approach (i.e., fitting a psychometric function in the case of the phoneme identification task). This reduced sample size reduces the statistical power. However, as can be seen in the recruitment flowchart in the supplementary material (S.1), we tried our best to get to a representative sample size of IWA (698 stroke patients were screened with a short language test in the acute phase), but there are also contextual factors and inclusion criteria that make it difficult to achieve a large sample size. Future studies with larger sample sizes may offer more insight into lower-level auditory processing in aphasia.

In conclusion, our results show that the RTD task and the phoneme identification task were each able to identify a processing impairment in 52-55$\%$ of IWA, whereas only 4–10$\%$ of healthy controls showed an impairment. At group level, performance on both tasks was lower for the aphasia group than for the control group. Moreover, we found that three-quarters of our aphasia sample do suffer from either acoustic or phonemic processing problems, in addition to potential phonological, semantic or syntactic processing impairments. Assessment of auditory processing is however currently not done in the clinic when it comes to diagnosing aphasia. Additionally, we demonstrated that performance on the RTD task predicted phonological processing skills. Future development of norm scores of the acoustic and phonemic tasks would allow to formally diagnose auditory spectro-temporal processing impairments in IWA and would thus help SLPs to target therapy towards those aspects. Both the acoustic and phonemic task only require a tablet and headphones and would thus be relatively easy to implement in the clinical context (hospitals, rehabilitation centra or SLP practices). Patients with aphasia in the acute, subacute and chronic phase after stroke that are testable for language tests are also able to perform these tasks, which do not require a verbal response from patients. Both tasks take between 5 and 10 minutes administration time and display the results immediately after completion. Due to their efficiency and feasibility, the RTD and the phoneme identification tasks may be useful for diagnosing lower-level auditory processing impairments and for therapy follow-up of aphasia in the clinical context. Further research would be required in order to validate the tasks and develop norm scores for both tasks.

## Supplementary Information

Supplementary Information 1.

Supplementary Information 2.

## Data Availability

All data generated or analysed during this study are included in this published article and its supplementary information files.
