# Supplementary material for: Acoustic and phonemic processing are impaired in individuals with aphasia
Source: Sci Rep. 2023 Jul 11;13:11208. doi: 10.1038/s41598-023-37624-w (PMC10336064; doi:10.1038/s41598-023-37624-w)
Supplement: Supplementary file 2 — Supplementary Information 2. [file 41598_2023_37624_MOESM2_ESM.pdf]

Supplementary material

Recruitment flowchart

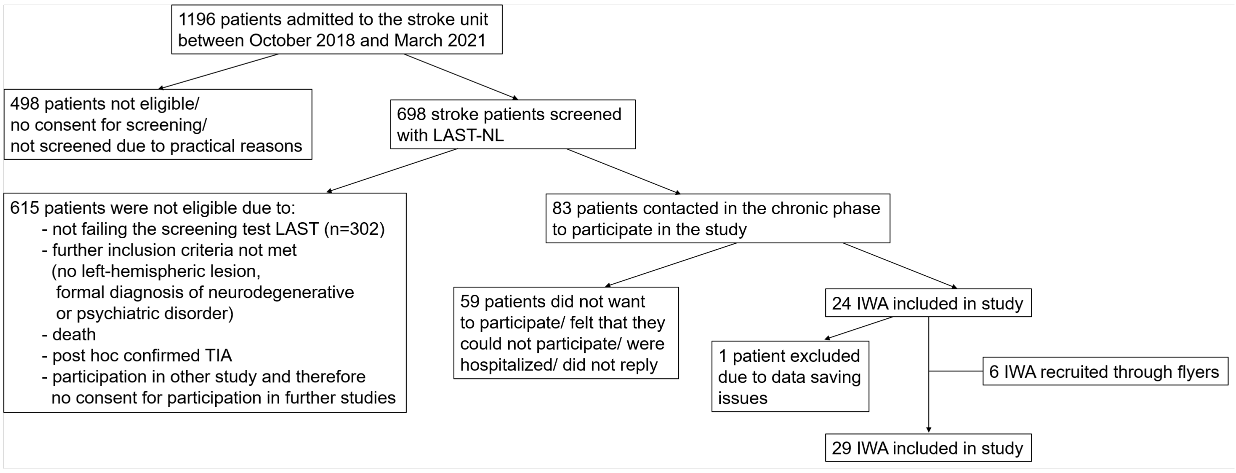

Figure S.1: **Flowchart of the recruitment procedure of individuals with aphasia as described in section 2.1 of the paper.**

Visualization of demographic and diagnostic variables

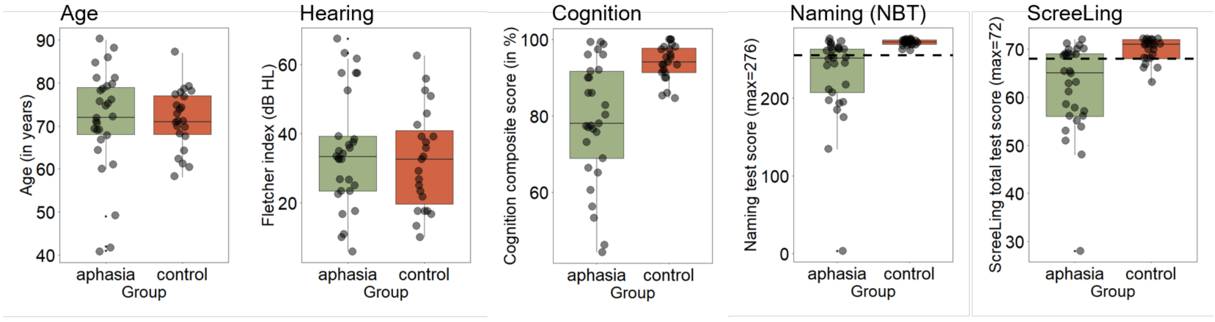

Figure S.2: **This figure shows demographic and diagnostic variables by group of the variables age, hearing, cognition, naming test and diagnostic language test.** The dashed lines on the two right most figures correspond to the cut-off threshold of those tests.

Hearing levels

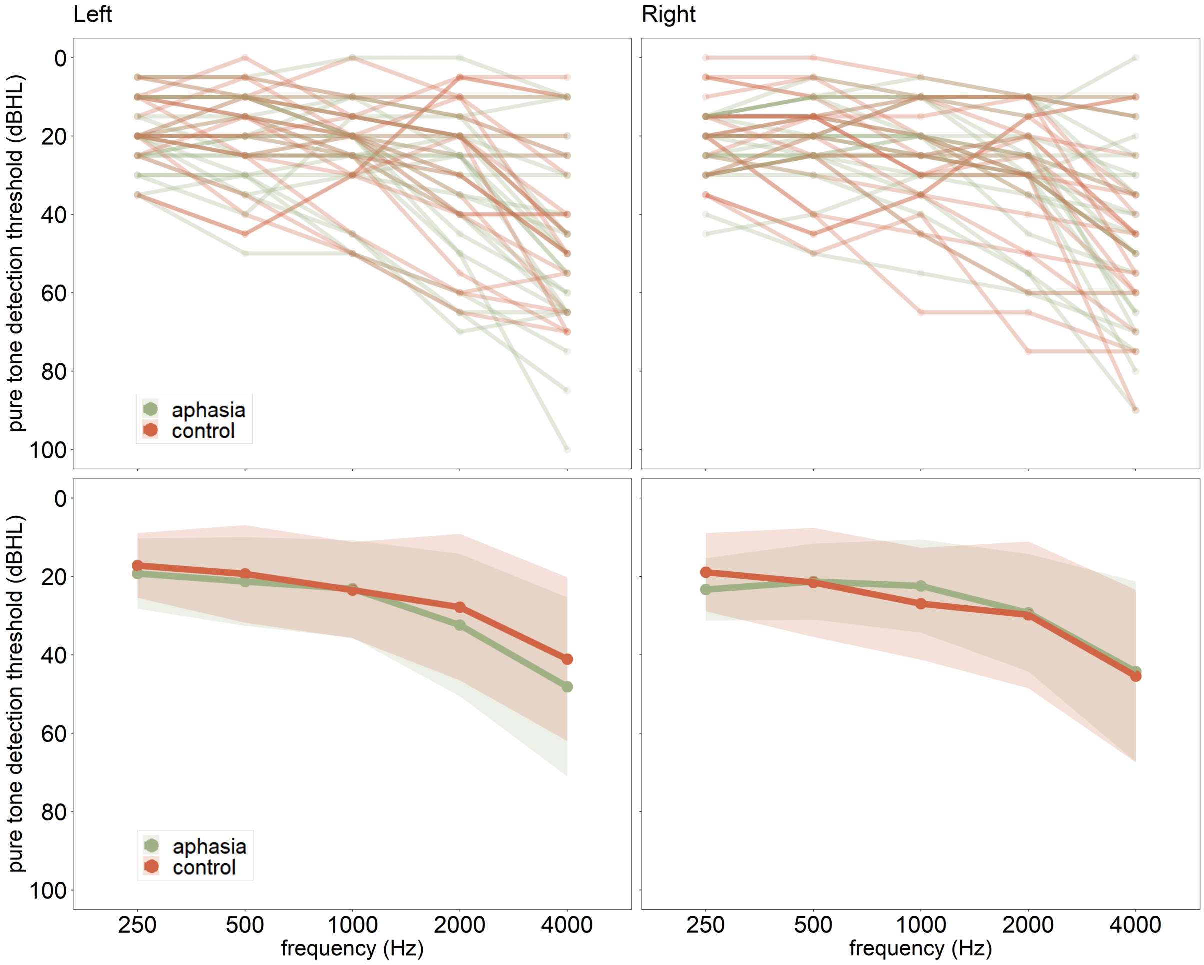

Figure S.3: **The pure tone audiograms by group.** The upper panels show individual pure tone detection thresholds for the left and the right ear respectively. The lower panels display the mean and standard deviation of pure tone detection thresholds by group for the left and the right ear respectively.

Correlations between psychoacoustic tasks and cognition

We report the within-aphasia group correlations between the psychoacoustic tasks and the cognitive score.

- RTD task $\sim$ cognition: Pearson's r = -0.43; p = 0.039 (The smaller the dicrimination threshold at the RTD task (i.e., the better the performance), the better the cognitive score.)
- Phoneme identification task $\sim$ cognition: Pearson's r = 0.04; p = 0.866

Education levels

Table S.1: Contingency table for education levels per group.

**Education level (years of education)**	**Aphasia group n (%)**	**Control group n (%)**
Primary school (6 years)	2 (6.90%)	0 (0.00%)
Secondary school (12 years)	10 (34.48%)	5 (21.74%)
College degree (15 years)	8 (27.59%)	8 (34.78%)
University degree (17 years)	9 (31.03%)	8 (34.78%)
Doctoral degree (21 years)	0 (0.00%)	2 (8.70%)

Normality assumptions of variables used for statistical analyses

Table S.2: Shapiro-Wilk and Levene's test results to check the normality assumptions.

**Effect**	**Test**	**W-value**	**F-Value**	**DF**	**p-value**
	**Rise time discrimination task**				
Group comparison without controlling	Normality (Shapiro-Wilk test)	0.96			0.205
	Homoscedasticity (Levene's test)		2.03	1, 44	0.160
Group comparison with controlling	Normality (Shapiro-Wilk test)	0.97			0.332
Phonology ScreeLing (within aphasia group)	Normality (Shapiro-Wilk test)	0.97			0.882
Phonological word fluency (within aphasia group)	Normality (Shapiro-Wilk test)	0.92			0.094
	**Phoneme identification task**				
Group comparison without controlling	**Normality (Shapiro-Wilk test)**	0.93			**0.031**
	Homoscedasticity (Levene's test)		2.33	1, 35	0.135
Group comparison with controlling	**Normality (Shapiro-Wilk test)**	0.91			**0.01**
Phonology ScreeLing (within aphasia group)	Normality (Shapiro-Wilk test)	0.96			0.658
Phonological word fluency (within aphasia group)	Normality (Shapiro-Wilk test)	0.96			0.694

DF = degrees of freedom; significant effects are marked in bold, meaning that the data are not meeting the normality assumption.

Individual deviance analysis

The individual deviance analysis allows to see which individuals with aphasia are deviant from the control group on the RTD task and the phoneme identification task (see paper for more details on the method). For the RTD task, the control sample was normally distributed after removing the lowest performing 5% ($\geq$ percentile 95) of the control group (Shapiro-Wilk normality test: W = 0.92, p-value = 0.122). For the phoneme identification task, the control sample was also normally distributed after removing the lowest performing 5% ($\leq$ percentile 5) of the control group (Shapiro-Wilk normality test: W = 0.95, p-value = 0.517). For the threshold estimation, all participant scores were standardized by subtracting the mean of the trimmed control sample and then dividing by the SD of the trimmed control sample. The deviance threshold was then defined at -1.65 SD (for the phoneme identification task) or 1.65 SD (for the RTD task) of the z-scored distribution.

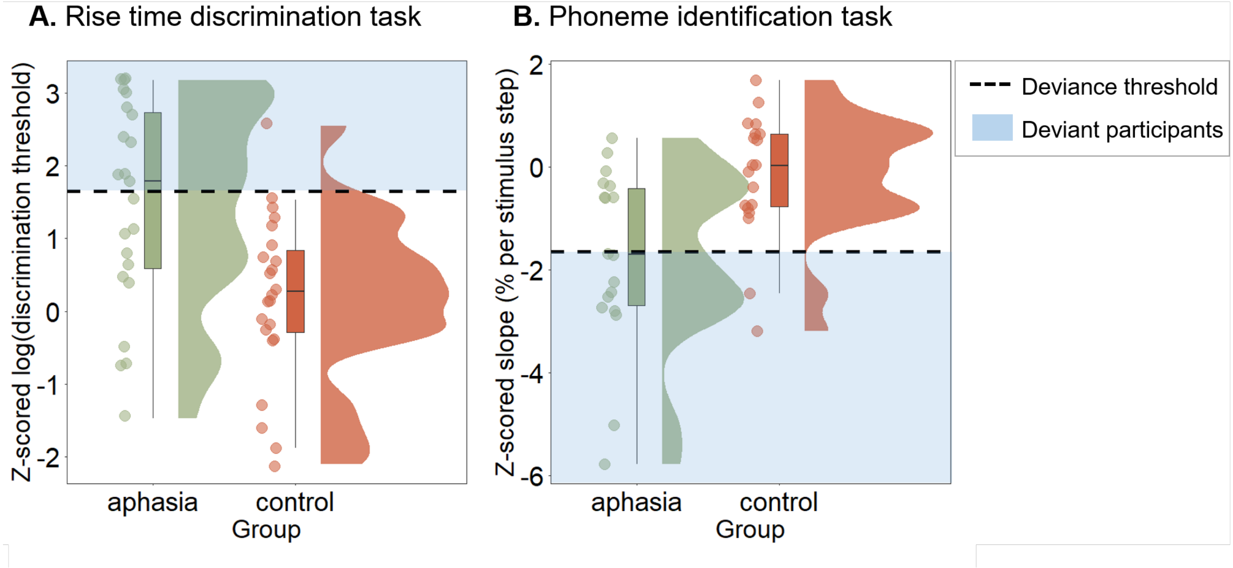

Figure S.4: **Individual deviance analysis of the acoustic and phonemic processing tasks. A.** Visualization of the deviant participants for the RTD task. **B.** Visualization of the deviant participants for the phoneme identification task.

Table S.3: Number of participants and percentage of deviance on the acoustic and phonemic tasks.

	**Aphasia group n(%)**	**Control group n(%)**
**Rise time discrimination task**		
deviant	12 (52.17%)	1 (4.35%)
not deviant	11 (47.83%)	22 (95.65%)
**Phoneme identification task**		
deviant	10 (55.56%)	2 (10.53%)
not deviant	8 (44.44%)	17 (89.47%)
**Overlap in deviance between the 2 tasks**		
overlap	6 (37.50%)	17 (89.47%)
no overlap	10 (62.5%)	2 (10.53%)
**Deviance on at least one of the 2 tasks**		
yes	19 (76%)	3 (13.04%)
no	6 (24%)	20 (86.96%)
